# Recognition of Reviewers

**DOI:** 10.1210/jendso/bvae011

**Published:** 2024-03-08

The Editors of *Journal of the Endocrine Society* thank all those who reviewed during 2023 for their time and effort.

David Abbott

Asmahan Abdalla

Yasmine Abdelmeguid

Hussam Abu Mousa

Julio Abucham

Adegbenga Ademolu

Sunita Agarwal

Andrew Agbaje

Benson Akingbemi

Dunia Alarkawi

Anastasia-Stefania Alexopoulos

Dalal Ali

G. Todd Alonso

Clara Alvarez

Ali Alzahrani

Sayeepriyadarshini Anakk

Bradley Anawalt

Greg Anderson

Siddhartha Angadi

Trevor Angell

Sonir Antonini

Helen Antony

Izzuddin Aris

Mai Arlien-Søborg

Decio Armanini

Ambika Ashraf

Richard Auchus

Laura Audi

Leon Bach

Ramiro Balderas

Eric Balti

Vaneeta Bamba

Irina Bancos

Amita Bansal

Giuseppe Barbesino

Alexander Bartelt

Shehzad Basaria

Ilana Bass

Rafael Batista

Rene Baudrand

Traci Bekelman

Giuseppe Bellastella

Jerzy Beltowski

Jerome Bertherat

Annabel Berthon

Traci Bethea

Matthias Betz

Maria Silvia Bianchi

Robert Blank

Sandra Blethen

Dana Bliuc

Zeev Blumenfeld

Richard Bockman

Anita Boelen

Matthew Brady

Maria Luisa Brandi

Gregory Brent

Vinicius Brito

Matthew Broome

Jenifer Brown

Eveline Bruinstroop

Luigi Brunetti

Raffaella Buzzetti

Michael Campbell

Irene Campi

Rossella Cannarella

Ana Canton

Massimiliano Caprio

Ruben Cardozo

Owen Carmichael

Denise Carneiro-Pla

Ty Carroll

Sally Carty

Melissa Cavaghan

Filippo Ceccato

Francesco Celi

Marco Centanni

Philippe Chanson

Dimitrios Chantzichristos

Lily Chao

Dong-bao Chen

Shanshan Chen

Weiwen Chen

Stanley Chen Cardenas

Ana Chiesa

Geoffrey Chilvers

Ken Chiu

Sophie Christin-Maitre

George Chrousos

Roderick Clifton-Bligh

Catherine Cohen

Annamaria Colao

Paulo Collett-Solberg

Carla Colombo

Fabio Comim

Rebecca Conway

Mark Cooper

Stephanie Correa

Rebecca Cunningham

Patricia Dahia

Neil Daniel

Feyza Darendeliler

Caroline Davidge-Pitts

Terry Davies

Shanlee Davis

Margaret de Castro

Carl De Crée

Miguel Debono

Angela Delaney

Daniel DeSalvo

Luigi Di Filippo

Beverly Diamond

Renuka Dias

Laura Dichtel

Johanna DiStefano

Jose Donato Jr.

Chrysoula Dosiou

Luciano Drager

Matthew Drake

Daisy Duan

Daniel Dumesic

Nancy Dunbar

William Duncan

David Ehrmann

Graeme Eisenhofer

Dariush Elahi

Thomas Fahey III

Heng-Yu Fan

Marina Fernandez

Maria Fleseriu

Christy Foster

Maria Candida Fragoso

Pamela Freda

Marie Freel

Peter Fuller

Jonathan Fussey

Thiago Gagliano-Jucá

Alexandra Gaspar

Luigi Gennari

Hans Ghayee

Andrea Giustina

Benjamin Glaser

David Goltzman

Celso Gomez-Sanchez

Anne Gompel

Robert Goodman

Geetha Gopalakrishnan

Andrea Gore

Daniel Gorelick

Caroline Gorvin

Ashley Grossman

Mathis Grossmann

Haixia Guan

Nese Ersoz Gulcelik

Dhananjay Gupta

Tulay Guran

Mouhammed Habra

David Handelsman

Sabine Hannema

Inga Harbuz-Miller

Rowan Hardy

Anthony Heaney

Emily Heiston

Peter Hindmarsh

Harry Hirsch

Thanh Hoang

Elizabeth Holt

Gregory Hong

William Horton

Sasha Howard

Ilpo Huhtaniemi

Gregory Hundemer

Jan Idkowiak

Peter Igaz

Maria Insenser

Brian Irving

Najmul Islam

Gabriel Jasul

Youn Hee Jee

Smita Jha

Gudmundur Johannsson

C. Christofer Juhlin

Takashi Kadowaki

Laura Kass

Leandro Kasuki

Megan Kelsey

Bernard Khoo

Rhonda Kineman

Gregory Kline

Georgeanna Klingensmith

Daniel Klink

Christian Koch

Lynn Kohlmeier

Josef Köhrle

Marta Korbonits

Anupam Kotwal

Lindsay Kuo

Enzo Lalli

Cristina Lamas

Nils Lambrecht

Michaël Laurent

Jennifer Law

Neil Lawrence

Jack Leahy

Phillip Lee

Richard Leslie

Angela Leung

John Lew

E. Michael Lewiecki

Xia Li

Chien Li

Chienying Liu

Zhenqi Liu

Kaitlin Love

Xiaoping Luo

Raul Luque

Steven Malin

Fernando Marin

Alejandro Martinez

Sarah Mayson

Gherardo Mazziotti

Shana McCormack

Janet McGill

Shlomo Melmed

Moises Mercado

Livia Mermejo

Takashi Minegishi

Dipika Mohan

Marie-Pierre Moisan

Mark Molitch

Christiaan Mooij

Terry Moore

Carla Moran

Rebecca Mountain

Paolo Mulatero

Ranganath Muniyappa

Madalina Musat

Angel Nadal

Endre Nagy

Kazutaka Nanba

John Newell-Price

Hannah Nieto

Natalie Nokoff

Jerome Nwachukwu

Gianluca Occhi

Onyebuchi Okosieme

Samuel Olatunbosun

Richard Oram

Julie Ostrander

Vasantha Padmanabhan

Purab Pal

Maria Papaleontiou

Theodora Pappa

Elizabeth Pearce

Luca Persani

John Phay

Sara Pinney

Rosa Pirchio

Melissa Premaor

Lauren Quinn

Hershel Raff

Ranjith Ramasamy

Sudhaker Rao

Aleksandra Rasic-Markovic

Rodolfo Rey

R. Paul Robertson

Vincenzo Rochira

Troy Roepke

Alan Rogol

Allen Root

Stephen Rosenthal

Susanne Rospleszcz

Alessandro Rubinacci

Yoshifumi Saisho

Nanette Santoro

Nicola Santoro

Maria Scarale

Nadia Schoenmakers

Carolyn Seib

Sabyasachi Sen

Joseph Shaker

Allison Shapiro

Neil Sharma

Praveen Sharma

Angela Sheu

Ilan Shimon

Dolores Shoback

Ashley Shoemaker

Leticia Silveira

Jennifer Sipos

Joel Smith

Daniel Spratt

Victor Srougi

Brendan Stack

Karmen Stankov

Devin Steenkamp

Insoo Suh

Larry Suva

Katrin Svensson

Antoine Tabarin

Yutaka Takahashi

Hesham Tawfeek

Peter Taylor

Anthony Taylor

Xiaochun Teng

Marily Theodoropoulou

Arthi Thirumalai

Wenxin Tong

Francesco Torino

David Torpy

Sulay Tovar

Lindsey Treviño

Nicholas Tritos

Stylianos Tsagarakis

Karen Tsai

Elena Tsourdi

Adina Turcu

Kohjiro Ueki

Catherine Van Poznak

A.S. Paul van Trotsenburg

Andre van Wijnen

Surendra Varma

Joseph Verbalis

W. Edward Visser

Gary Wand

Pandora Wander

Tracy Wang

Genoa Warner

Nelson Watts

Ram Weiss

Robert Wermers

Stephen Winters

Joseph Wolfsdorf

Denise Wong

Chih-Hsing Wu

Tao-Yiao Wu

Carol Wysham

Catherine Yeckel

Bulent Yildiz

John Yoon

Kevin Yuen

Henry Zelada

Jie V. Zhao

Yi Zheng

Zhengui Zheng

Kun Zhu

R. Zoeller

